# Cerebro-Spinal Meningitis, with a Report of Cases

**Published:** 1866-06

**Authors:** W. S. Armstrong

**Affiliations:** Atlanta, Ga.


ATLANTA

### Medical and Surgical Journal.

NEW SERIES.

VOL. VIIB JUNE, 1866. No. 4.

### ORIGINAL COMMUNICATIONS.

### ARTICLE I.

*Cerebro-Spinal Meningitis, with a report of Cases.* By W. S. ARMSTRONG, M. D., of Atlanta, Ga.

In general, the symptoms which characterize this disease are of a severe character. In the midst of good health, after taking a hearty meal, or after a full day's work, the patient, without any premonitory symptoms, is suddenly attacked with coma, or stupor, so profound that he is with difficulty aroused even for a moment.

In other cases vertigo, pain in the head and cerrical region, extending along the spine, with lassitude and apprehension of impending danger are observed. Then again, chilly sensations at intervals of two or three hours, with cold extremities, followed by exacerbations of heat, flushed face and increased pulse, mark the approach of the disorder. Lastly, delirium, more or less wild, with a disposition, forcibly, to leave the bed or room, is in the outset a prominent symptom.

The epidemic of Cerebro-Spinal Meningitis, as it came under my observation at Mobile, Ala., during the winters of 1863–'64, and '64–'65, no matter what the symptoms, either in the formation or advanced stage, was equally grave in its import.

The condition of the pulse was variable; usually ranging from ninety to one hundred, hardly reaching one hundred and ten, unless just before the termination in death; on the other hand, it occasionally sank to forty or fifty beats per minute. Vomiting of bile and constipation are usually in the beginning, prominent symptoms; the tongue is furred, and as the disorder advances, the teeth become covered with sordes.

The urine is highly colored, scanty and often retained; at other times, especially towards the close, it is passed involuntarily.

Intolerance of light and sound, when present, appear at the early part of the attack—the least ray of light being sufficient to cause spasmodic closure of the eyes and intense suffering; walking across the floor is excessively annoying to the sufferer; deafness and a general indifference to surrounding objects, is occasionally noticed.

The most prominent and almost universal symptoms, are pain in the head and neck, accompanied by a tetanic rigidity of the cervical muscles, and of the large extensor muscles of the back. This trouble, slight at first, increases until the head is drawn back upon the shoulders, and no ordinary degree of force used by the attendant can overcome it. The muscles of the back and lower extremities are occasionally so much involved as to produce complete opisthotonos. In connection with this condition, paralysis of the muscles of the face is sometimes present, as exhibited in depression of the lower jaw and protrusion of the cheeks and lips in expiration. Involuntary twitchings of the muscles and want of prehension often exist also—the patient being unable to drink without assistance. Strabismus in one or both eyes was met with in several cases. The appearance of the pupils is not always the same, in the majority of cases being dilated; sometimes one is contracted and the other dilated, and I have occasionally seen both contracted.

Delirium may be present at any period of Cerebro-Spinal Meningitis, though most common in the latter stages before coma sets in, and is then of a low, muttering character.

When coma comes on, which is usually about the fourth or fifth day, the pupils become widely dilated, the pulse more full, but is never, so far as my observation extends, of a bounding character, as in coma from apoplexy. Involuntary discharges from the bowels and bladder are now of most frequent occurrence. Stertorous breathing is rarely present, and until the coma is profound, the patient is continually tossing himself from side to side in bed, and carrying his hands to his head as though in great pain.

Another very common symptom is Hyperœsthesia of the whole nervous system; pressure upon the extremities, slight moving of the feet or bending the toes, causes the patient to cry out from pain. This exaltation of sensibility does not often appear at first, but towards the latter part of the attack. While vertigo, pain in the head, chilly sensations, intolerance of light and sound, deafness, stupor, exalted sensibility of the nervous system, delirium and coma were the usual symptoms by which this epidemic was characterized, yet there were a few cases of an intermittent type, accompanied by high fever with pain in the head. Under the use of Quinia these symptoms would yield for a few days and convalescence seemed to be established. A recurrence of these symptoms would take place two or three times, when those more violent, as extreme pain in the head and neck, rigidity of the muscles, &c., would supervene, and declare unmistakably the formidable nature of the disease.

The duration of this affection is variable; it may destroy life in twenty-four or forty-eight hours, but from five to eight days is the usual time. During the winter of 1863-64, it proved fatal sooner than in the following—a few of the last cases seen having lived from ten to fifteen days.

Case 1.—*Cerebro-Spinal Meningitis—Effusion of Lymph on Cerebrum and at the base of brain—Serum in lateral ventricles.*—Private H. C. ——, age about forty, was admitted into hospital, Jan. 17, 1865. Antecedent history. Some six weeks ago, after being exposed to the weather a good deal, both day and night in camp, he had three chills, the last of which was congestive, and four days elapsed before consciousness returned. In a short time he was convalescent. This history was obtained from a comrade. Present condition. He complains of soreness from head to foot, has frequent rigors, and is unable to express himself clearly; pulse 90; tongue moist and furred; bowels constipated. Ordered a cathartic and Quinia. Jan. 18.—Pulse 93; bowels moved five times from purgative; skin has been warm since admission; pain in the head and neck. Ordered blister to the back of the head and neck; repeat Quinia. Jan. 19.—Pulse 90; pupils contracted; unconscious; head drawn back from contractions of muscles; breathes through his mouth; raises his hands to his head as though in pain. To have blister to the head. Jan 20th.—No improvement; pulse 100 and weaker. To have cathartic and spirits of wine at short intervals. Jan. 21.—Pulse 120 and very feeble; skin warm and perspiring; comatose; opisthotonos, which began to develop itself yesterday, is very marked. Died at 11 P. M.

Autopsy Jan. 22d. The anterior, two-thirds of cerebrum superiorly, are covered with an adventitious deposit of lymph, of a greenish yellow color, forming adhesions between the arachnoid and pia mater, and following the latter as it dips down into the convolutions of the brain. The under surface of the anterior lobes, optic commissure, crura cerebri and pons varolii, are the seat of exudation also. Some pus is found at the medulla oblongata. This exudation is from one to two lines in thickness. The lateral ventricles are distended with effusion, which being drawn off, pus is found at the bottom. The choroid pexus is injected. The brain shows, on section, no indurations or softening, but appears healthy.

Case 2d.—*Cerebro-Spinal Meningitis—Effusion of Serum in Arachnoid space—Deposits of Lymph on brain and Spinal-cord.*

Private W. F. D—— was admitted Jan. 19, 1865, at 10 A. M. Present condition. Pulse 80; skin moist and cool; pupils natural; restless; stares at his attendants; makes no reply to questions addressed to him; his cheeks protrude at each expiration. To have Quinia. Jan. 20th.—Pulse 98; had several actions on bowels; pupils respond to light feebly; both wrists swollen and painful to the touch; notices what is going on in the room, but seems to be unable to articulate. Continue treatment. Jan 21st.—His mind is clearer; answers some questions rationally; for the first time, he protrudes his tongue, which is furred and red; took some nourishment; bowels acted last night; picks at his bed clothes; has passed no urine since yesterday; one pint is drawn off. Continue treatment.

Jan. 22d.—Has been perspiring freely for several hours; delirious; head is drawn back and to the right side; has had rigors since yesterday, at intervals; no action on bowels; drew off two pints of urine; ordered head to be shaved and a blister applied; also a purgative. 3 P. M.—Pulse 140 and irregular; pupils contracted slightly; right eye drawn outwards; comatose; no action on bowels; drew off one pint of urine. Died in the early part of the night.

Autopsy, Jan. 23d. Effusion of serum in the arachnoid cavity, considerable; membranes very much injected; slight deposit of lymph between the pia mater and arachnoid, on the anterior surface of the cerebrum superiorly, extensively upon and around the optic commissure, over the entire cerebellum, crura cerebri, pons varolii, medulla oblongata and spinal cord, throughout its whole extent to the cauda equina. The nerves arising from the cord on both sides were enveloped with this deposit also. At several points along the cord it had degenerated into pus. The abdominal viscera upon examination appeared normal. The lungs were healthy also. The right side of the heart was distended with blood, and the right ventricle contained a clot of fibrin occupying half of its chamber.

Case 3d.—Private F. G—— was admitted Feb. 3, 1865. He is suffering severe ptyalism from Calomel, which he took on his own account; his skin is yellow; complains of lassitude; pulse natural; has vomited bile several times; he is allowed to go into private quarters; to have mouth wash. Feb. 4th.—Was sent for to see patient this morning; found him suffering from pain in the frontal and temporal regions, coming on late last night; tongue swollen and ulcerated; has rigors occasionally; vomited bile during the night and this morning; no action on bowels. To have Quinia and Opium, and a cathartic. Feb. 7th.—Rested very little last night; complains of severe pain in the head; at times his mind is wandering; rises up in bed frequently and attempts to leave the room; had four actions on bowels last night; continue treatment. Feb. 8th.—Slept none during the night; has violent pain in the head; is more rational. Continue Quinia. At 1 o'clock, P. M., was called to see patient; found him delirious; complaining much of pain in the head and along the spine, which was tender on pressure. To have cold applications to head, and blister to spine; also hot foot bath; pupils a little dilated. Feb. 9th.—Pulse still natural; skin warm; pupils dilated; bowels acted freely; passed urine; the extremities very sensitive to the touch; can't bear the weight of the bed clothes; delirious; picks at his bed; refuses nourishment; mouth and tongue very sore. To have Anodyne to secure rest; large doses of Iodide Potass. frequently during the day; spirits of wine four times a day. Feb. 10th.—Pulse weaker; slept three hours during the night; more rational; complains of pain at the back of his neck when his head is moved. Continue treatment. Feb. 11th.—Pulse 110, and very weak; pupils sluggish; delirious during most of the night; pain in the head; he is growing weaker; unable to clear the air passages of mucus; to have spirits of wine freely. 5 o'clock, P. M. Pulse 150; sinking rapidly. Died in the early part of the night. No autopsy.

CASE 4th. *Cerebro-Spinal Meningitis.—Deposit of Lymph superiorly and at the base of brain.—Serum in the Arachnoid Cavity.*

Private W. J. H—— was admitted on the 8th Dec., 1864. History: The Surgeon of his regiment informed me that he was found in bed this morning, apparently suffering from congestive fever; the attack came on while asleep. Present condition: He is unconscious; pulse 119; pupils dilated; skin cool. To have stimulants internally and frictions with Spirits of Turpentine; also blister to head and spine. Dec. 9th.—Pulse 100, and increased in volume, but still not strong; teeth covered with sordes. Continue treatment. Dec. 10th.—Pulse 120, and feeble; skin cool; comatose. Died at 10, A. M.

Autopsy, Dec. 11th. Effusion in the arachnoid space. On the anterior surface of cerebrum superiorly, there is a deposit of adventitious membrane, also on its under surface as far back as the crura cerebri. The cerebellum below has a deposit of the characteristic greenish yellow appearance.

The prognosis in this epidemic was very unfavorable; in fact, it was regarded a death warrant to the subject. I can safely say that notwithstanding my observations were extensive among the soldiers and negro laborers in and around Mobile, I never saw a single case recover. Indeed, we would ask, when such pathological conditions as universally presented themselves, affecting the meninges of the brain and spinal cord, are present, what other results could be expected?

In the two varieties of cases, which were observed, a treatment supposed most likely to succeed was adopted. In the one where there was much febrile excitement, antiphlogistics were used—in the other and opposite condition, a supporting plan was followed, and both alike, with unfavorable results. Quinia in large doses in previous epidemics was found to relieve a certain per centage of those attacked, but in this, it failed in doses from a scruple to a drachm. So likewise, chloride of Mercury was found to act beneficially, but here, it failed also, even after its specific effects were produced, as was exemplified in Case No. 3, where it was accidentally brought about, before the disease made its appearance.

Before pain in the head and neck, rigidity of the muscles and coma are developed, we have no diagnostic signs to justify active measures, and after they occur, effusion of lymph has taken place, and, if in a sufficient amount to suspend the functions of the brain and spinal cord, the result, it seems to me, is inevitable, and little or no good, with the present lights before us, can be expected from treatment. Yet, I have thought and still think, that in mild cases where the functions of nutrition and decrition could be performed for a sufficient length of time, though imperfectly, under a supporting plan of treatment, nature would restore a healthy condition of the cerebro-spinal centers by a breaking down and absorption of the effusion. This favorable result we meet with in pneumonia, where the lungs are the seat of the effusion. Re-assuring from induction, this mode of treatment seems to offer most hope of favorable results.

